# Capacity of community advisory boards for effective engagement in clinical research: a mixed methods study

**DOI:** 10.1186/s12910-021-00733-0

**Published:** 2021-12-15

**Authors:** Levicatus Mugenyi, Andrew Mijumbi, Mastula Nanfuka, Collins Agaba, Fedress Kaliba, Irene Seryazi Semakula, Winfred Badanga Nazziwa, Joseph Ochieng

**Affiliations:** 1 The AIDS Support Organization, Kampala, Uganda; 2 Makerere University-John Hopkins University Collaboration, Kampala, Uganda; 3 Uganda National Council for Science and Technology, Kampala, Uganda; 4 College of Health Sciences, Makerere University, Kampala, Uganda

**Keywords:** CABs, Capacity, Research communities, Community engagement, Research ethics, Training, Knowledge

## Abstract

**Background:**

Community engagement is a key component in health research. One of the ways health researchers ensure community engagement is through Community Advisory Boards (CABs). The capacity of CABs to properly perform their role in clinical research has not been well described in many resource limited settings. In this study, we assessed the capacity of CABs for effective community engagement in Uganda.

**Methods:**

We conducted a cross sectional study with mixed methods. We used structured questionnaires and key informant interviews (KII) to collect data from CAB members, trial investigators, and community liaison officers. For quantitative data, we used descriptive statistics while for qualitative data we used content analysis.

**Results:**

Seventy three CAB members were interviewed using structured questionnaires; 58.9% males, median age 49 years (IQR 24–70), 71.2% had attained tertiary education, 42.5% never attended any research ethics training, only 26% had a training in human subject protection, 30.1% had training in health research, 50.7% never attended any training about the role of CABs, and 72.6% had no guidelines for their operation. On the qualitative aspect, 24 KIIs cited CAB members to have some skills and ability to understand and review study documents, offer guidance on community norms and expectations and give valuable feedback to the investigators. However, challenges like limited resources, lack of independence and guidelines, and knowledge gaps about research ethics were cited as hindrances of CABs capacity.

**Conclusion:**

Though CABs have some capacity to perform their role in the Ugandan setting, their functionality is limited by lack of resources to facilitate their work, lack of independence, lack of guidelines for their operations and limited knowledge regarding issues of research ethics and protection of the rights of trial participants.

**Supplementary Information:**

The online version contains supplementary material available at 10.1186/s12910-021-00733-0.

## Introduction

Community engagement is a key component in health research as it creates a platform where the researchers meet with the community thus creating a common understanding for the two parties. Community Advisory Boards (CABs) is one of the approaches that has been used especially for HIV clinical trials to engage the community in health research [1]. A CAB can be defined as a group of people that formalizes the institution–community partnership and guide the research by providing a mechanism for community members to have representation in research activities [2]. As a principle of good research practice, all clinical trials are expected to have a CAB to work as a link between the clinical trial’s team and the community where the study is being implemented [3]. This engagement ensures that the community has input into the research process from design to implementation and dissemination of findings. Uganda formed one of the first CABs in Africa in the 1990s in conjunction with an HIV vaccine project [4].

CABs are not responsible for scientific decisions or ethical protection of subjects but can provide input into informed consent, educational materials used in the trial, input on issues of cultural sensitivity and appropriateness of incentives given in the trial [5, 6]. CABs play an important role for helping researchers to better understand the community at each phase of the clinical trial [7], hence, building mutually beneficial relationships between the researcher(s) and the communities in which research is being implemented [8].

In Uganda, CABs are widely used by most research institutions and this has enabled researchers to gain entry into the different research communities. The CABs play the role of community consultation and sensitization as well as ensuring that the rights of the research participants are respected and protected. However, for the CABs to effectively play this role, the members have to understand the principles of community engagement and ethical conduct of research, the study protocol and social-cultural dynamics of the planned research communities.

The capacity of CAB members to understand research ethics, identify potential harms to community and communicate feedback to researchers remains critical to optimize engagement of lay communities [9]. CAB members perceive their consultations to be helpful in improving the capacity of researchers and the quality of research projects [10]. However, several challenges have been cited to limit the functionality of CABs. These include incomplete ethical regulations and guidance; limited knowledge of science among members of communities and CABs; poor CAB management; language barriers between research staff and community members [11]; and CAB independence due to support provided by the research team in the form of transport reimbursements and other forms of support [12].

Generally, there is a paucity of evidence on the capacity of CABs in Africa and in Uganda in particular regarding the extent to which they can perform their roles in different research settings. Assessment of CAB’s functioning has not been well-established. Guidelines prescribing the formation and institution of CABs do not specify a formal methodology of how they should function. In Uganda, this is partly due to limited guidance from the official regulator of research activities in the country—the Uganda National Council for Science and Technology (UNCST) [13] on how such CABs can operate, and what skills and training they require to properly perform their role. The establishment and operation of CABs in Uganda is currently a responsibility of the research institutions, or principal investigators [13] who usually focus on training CAB members about the research protocols and less on giving skills and training that can enable them to properly perform their role. This consequently results in limited knowledge on functionality of the CABs.

In this paper we aimed to assess the capacity of the CABs in performing their role, and identify knowledge and training gaps for the CAB members in Uganda.

## Methods

### Study design and setting

We used a cross-sectional design with mixed methods approach of concurrent data collection and analysis. The study was conducted in Uganda between March and October 2020 covering 19 research institutions that were conducting clinical trials according to the National Drug Authority (NDA) database [14]. We randomly selected 26 clinical trials out of the 74 that existed in the database including both completed (not more than one year) and ongoing trials. We randomly selected 1 clinical trial per institution conducting 1 to 4 trials, 2 clinical trials per institution conducting 5–9 trials, 3 clinical trials from each institution conducting 10–14 trials, and 4 clinical trials from institutions conducting 15 or more trials. This resulted into a total of 26 clinical trials from the 19 research institutions. All CABs attached to the selected clinical trials were included in the study. The study participants included CAB members, CAB chairpersons, trial investigators, and community liaison officers. A community liaison officer is a person based at the research institution and his/her role is to link the research institution or investigators to the communities where they wish to conduct the study.

### Data collection

For the quantitative component, we conducted face-face interviews with CAB members using a structured questionnaire. We used an open source software “KoBo Toolbox” to design an electronic version of the questionnaire and installed it on tablet computers which were then used to collect field data. Every day, the research assistants uploaded the collected data to the server supported by the “KoBo Toolbox” platform enabling timely reviewing and cleaning of the data. At the end of the data collection exercise, the data were downloaded from the “KoBo Toolbox” platform and exported to STATA software for further management and analysis.

For the qualitative component, we used key informant interviews (KIIs) with the trial investigators, CAB chairpersons and community liaison officers. We considered a purposive sample of individuals who had a good understanding of community engagement in clinical trials in Uganda. A KII guide was designed and utilized during key informant interviews. The guide included questions on role of investigators and CAB members in clinical trials; challenges of community engagement; facilitation of CABs; regulatory oversight of CABs; work relationships between investigators and CABs; and opinions on how community trials should be conducted among others.

### Data analysis

For quantitative data, we used frequencies and proportions expressed as percentages to summarize categorical variables and median to summarize continuous variables. For the qualitative component, the collected data were transcribed verbatim and translated into English from local languages which included Luganda, Runyankole, Lusoga and Lusamia. A code book was later generated using the English transcripts. Thematic analysis was used to analyze the data based on emerging themes in line with the study objective. Qualitative information is presented as narratives and quotes. STATA version 14 [15] and Atlas.ti [16] computer software were used to analyze quantitative and qualitative data, respectively.

## Results

For the quantitative study, a total of 73 CAB members were contacted and they all participated in the individual interviews. Of these, 43 (58.9%) were males. The median age was 49 [interquartile range (IQR) 24–70] years. Table 1 shows the distribution of the socio-demographic characteristics of the CAB members.

**Table 1:** Distribution of demographic characteristics of CAB members (N = 73)

Characteristics	Number	Percentage (%)
Sex		
Female	30	41.1
Male	43	58.9
Age (years)		
< 30	6	8.2
30–39	11	15.1
40–49	23	31.5
50–59	21	28.8
60–69	11	15.1
70+	1	1.4
Highest level of school completed		
None	1	1.4
Primary	3	4.1
Secondary	17	23.3
Tertiary/university	52	71.2
Main occupation		
Formal employment	10	13.7
Farming	13	17.8
Business	45	61.6
Other	5	6.9
Marital status		
Married/co-habiting	54	74.0
Widow/divorced/separated	7	9.6
Never married	12	16.4

**Fig. 1:**
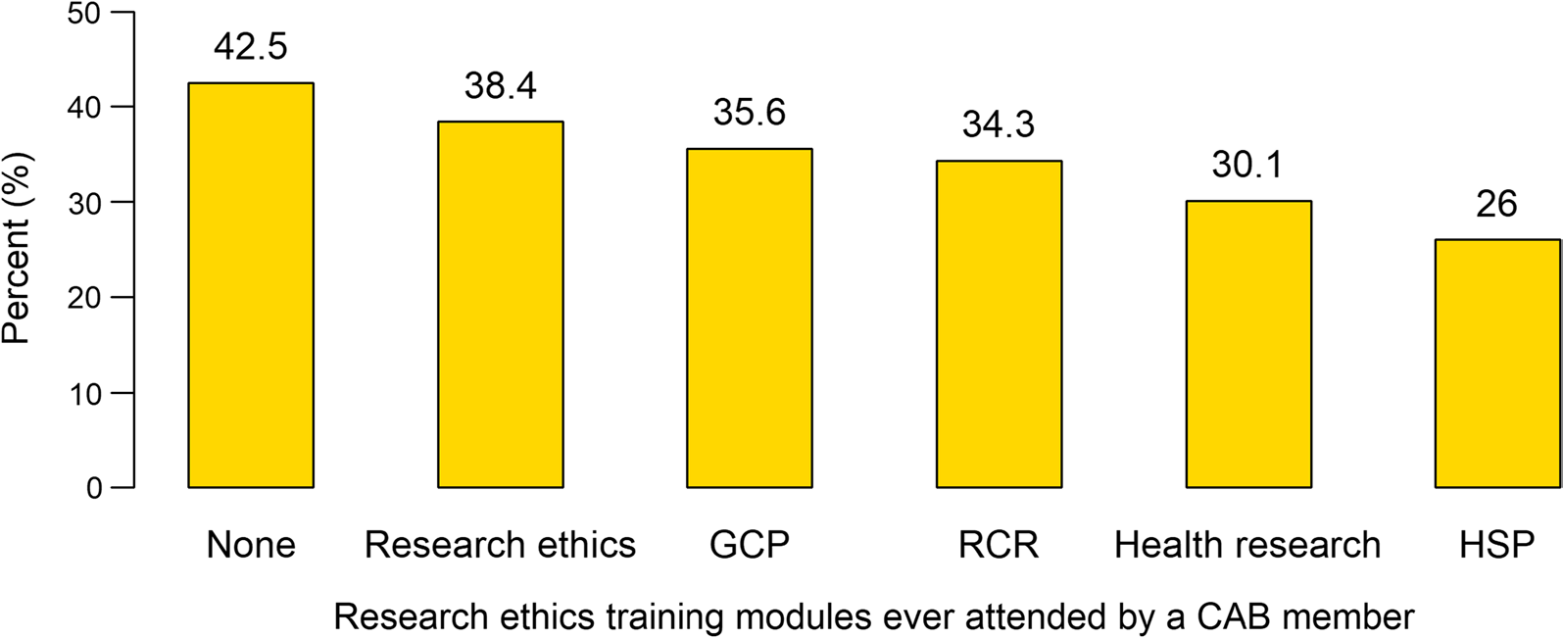
Percentage distribution of CAB members and the ethics training attended

Of the 73 CAB members surveyed, 42 (57.5%) said they did not attend any research ethics training module. Figure 1 shows the proportion of CAB members who reported to have attended the following courses: research ethics, good clinical practices (GCP), responsible conduct of research (RCR), health research, and human subject protection (HSP).

Of the 73 CAB members, 50.7% reported to have no training on CABs operations. Of those who had at least one training, 63.9% had the ethics training initiated by the trial investigators as shown in Table 2.

**Table 2:** Training of CAB members

	Number	Percentage (%)
Number of times CAB members reported to have attended training on CABs operation during the period they served as members	N = 73	
Never	37	50.7
Once	6	8.2
Twice	12	16.4
Three or more	18	24.7
Who initiated and facilitated the training	N = 36	
Trial investigator	9	25.0
Research institution	23	63.9
Regulatory body e.g. UNCST, IRB	1	2.8
Others	3	8.3

For the qualitative study, key informant interviews were conducted with 10 CAB chairpersons (female: 5); 10 trial investigators (female: 3); and 4 community liaison officers (female: 2). The distribution of KIIs by sex and age is shown in Table 3.

**Table 3:** Distribution of sex and age of participants for the qualitative component (KIIs)

Characteristics	CAB chairpersons	Trial investigators	Community liaison officers
	N = 10	N = 10	N = 4
Sex, n (%)			
Male	5 (50%)	7 (70%)	2 (50%)
Female	5 (50%)	3 (30%)	2 (50%)
Age (years)			
Median (IQR)	48 (47, 58.8)	40 (34.8, 47)	44 (39.3, 47.3)
Min, max	32, 75	31, 62	28, 54

Content analysis of the qualitative data identified three themes including (1) perspectives of researchers on CAB's roles in research, (2) the need for training and (3) challenges faced by CABs. Below we present results from the content analysis by these themes.

### Perspectives of researchers on CAB's roles in research

Respondents noted that CAB members had the skills and ability to understand certain study documents such as the consent forms, the interview instruments, information sheets targeted to the participants and they would therefore, support in the review of these documents and advise the research team accordingly. Different investigators acknowledged that CAB members were supportive in their efforts to have their study materials tailored to their target communities and they were also able to give relevant feedback to the study team on different issues regarding the study.

In most cases they also help us soften the language. For example, these trials are so technical; the language is so technical. For instance, when you talk about randomization, we sit with these ordinary people drawn from the community where we work and then they advise us on how best we can explain this concept of randomization. I remember some time back the first clinical trial we did, double blinded is a concept that I didn’t know how to put across to the communities. But when we met with the advisory board, they said instead of using a single word “kantuntunu” describe the process; describe the rationale behind double blinding of both the volunteers and then the scientific value in the randomization and blinding. And once we did that, then people understood. So that is the primary role that community advisory board members play (KII with Community Liaisons Officer).

We do receive a lot of feedback from them (CABs) because whenever we present to them, they give us quite a number of things; what ideally a person who is not a scientist would have not thought about in a study. So, I think they are very useful; they give very good feedback that is very important in the follow up of these participants and also as we implement the study. They do give very valuable feedback (KII with Trial Investigator).

### Need for training

Qualitative findings highlighted some gaps in training of CAB members. The investigators mainly focus on training the CAB members on the study protocol and less on general research principles. The training usually includes a lay orientation to the research process and community engagement procedures specific to the trial and this happens at the onset of implementation.

Apart from training them on the protocol, we haven’t yet given them a proper training. There are a number of trainings that can be given to them including research in general but we have not done that yet. As a team, it is a wakeup call for me to plan and have some engagements and train them (KII with Trial Investigator).

I don’t remember any trainings that we have conducted for our CABs apart from those mini trainings or meetings like the very first CAB meeting where we tell them about their terms of reference, what is expected of them and what is expected from the study team. And then the refresher in the middle so they can always remember their roles (KII with Trial Investigator).

We usually give them an overview about the study, target participants, the eligibility and also ask for their input. We also train them on the planned community engagement activities and then we shall need the CABs especially in recruitment and following up participants (KII with Trial Investigator).

Other respondents alluded to the fact that CAB members should get trained on other issues important in the conduct of clinical trials such as research literacy and advocacy in community engagement in trials. This however, was often tailored to the interests of the research team in respect of the study they are undertaking.

First, we make them aware and appreciate the role of research in our day to day life, basically research literacy. The second is advocacy- how to engage the communities; and then, their roles and responsibilities as a community advisory member, their mandate (KII with Trial Investigator).

It was noted that in order to ensure CAB members are adequately empowered to effectively perform their roles, trainings and capacity building for CABs should not be primarily left to the trial investigators and research team. Other stakeholders such as the research regulatory bodies and ethics committees should take up the role of giving capacity to CABs and empower them as required.

In a training session you may mention one or two ethical issues but spend more time on the project and the integrity. That role shouldn’t be left to the Investigators. Ethical training for the CABs should be a role for the REC (KII with Trial Investigator).

### Challenges faced by CABs

Whereas our discussions with CAB members revealed that a section of them believed they were performing their roles as expected, other CAB members admitted that they were not as effective as they are portrayed to be. It was reported that CAB members had challenges comprehending certain scientific concepts (such as blinding, placebo, treatment arm, intervention arm and randomization) involved in certain clinical trials.

We need the PIs to support the CAB. We need more relevant information on trials. As CAB members, we need to understand the research language. For example, you are talking about placebo but there are so many members you can ask about placebo and they don’t even know what it is. We need to get to a level where we understand things (KII with CAB Chairperson).

Following a power point presentation, a Professor challenged one of the technical staff on the study and said, “thank you for your presentation but isn’t this too big for these people?” Looking at the composition of our CAB, I am a reverend, the other one is a sex worker. What they need to do is to simplify the information in that language you understand but not the scientific terminologies which confuse us and opt out of the CAB meeting (KII with CAB chairperson).

The other challenges cited to negatively impact on CAB members’ effectiveness included; lack of independence, lack of necessary guidelines for CABs functioning, limited resources and funding to facilitate CAB’s work, CABs having to participate in different trials concurrently, knowledge gaps about research ethics and the rights of trial participants. The other concerns raised were: lack of basic training in research and limited time as many CAB members do go about their other livelihood activities to fend for themselves and their families.

Theoretically they [CABs] are independent but practically they are not (KII with Trial Investigator)

There should be a level of independence for them to do and say what their conscience tells them without fearing any sort of reprisal from the research team. They should be in a way that they can do their work without fear or favor and they are respected and it doesn’t put them in any jeopardy and stand for the community as they do for research (KII with Trial Investigator)

The CAB is a ghost committee; that is what I must tell you (laughs). The CAB committee is there in documents, but they don’t know the work for which it was instituted. And out of all these trial participants, you have invited today, if you asked them who the CAB members are they will not tell you and some of them have never heard about the word CAB (KII with Community Liaison Officer).

## Discussion

The study set out to assess the capacity of CABs for effective engagement of communities during conduct of clinical trials. Our study offers a documentation of the capacity of CABs and various challenges that affect the functionality of the CABs in resource limited settings. The findings show that CAB members have some level of capacity to understand and review study documents and can offer guidance on community norms and expectations as well as giving valuable feedback to the investigators and relay back to communities. However, there are some gaps in knowledge and skills that hinder their functionality. For example, a large proportion of CAB members (42.5%) had not attended any research ethics training, only a quarter had training in human subject protection, and more than half had never attended a training about the role of CABs. There are also other several challenges that were cited to be limiting their capacity. These include: limited resources to facilitate their work; lack of independence; lack of guidelines for their operation; and inability to understand and explain certain scientific concepts like placebo and randomization.

CABs’ roles involve capacity to understand the trial, review study documents, and provide input on community norms and values, which were highlighted in the findings, though not adequately addressed because of the cited challenges. Thus, for CABs to effectively perform their roles, such challenges need to be addressed. Lack of resources to enable the CABs to operate well may lead to inefficiency in performing their roles. This means that CABs will not be able to hold meetings, effectively engage with communities and will be unable to perform independently.

CABs are appointed and facilitated by the investigator and or research institutions and in so doing they are expected to pay allegiance to the investigator. This apparent lack of independence may compromise them to perform their roles because they may choose to be answerable to the researchers rather than serve the interests of the community. Thus, CAB members may not report any observed trial violations to any other authority other than the researcher or the research institution which may result in minimal protection of the trial participants. Some of our findings emphasize results from other settings. For example, some previous studies also reported CAB independence [1, 12] and this has been attributed to the support provided by the research team in form of transport reimbursements and other forms of support [12]. The lack of independence of CABs can be minimized if their operation is regulated by the national regulator of research activities. By so doing, the CABs will have the power to directly report any violations to regulatory bodies like the ethics committees other than to only the investigators.

Inadequate training to CAB members makes it difficult for them to properly explain research concepts like placebo, randomization or blinding, informed consent as well as rights and welfare of research participants hence rendering them ineffective. The gap in training of the CABs reported in our study further supports the work by Mlambo et al. [1], where they recommended for the provision of continuous training and capacity building of CABs if we are to impact on their functioning. However, the studies described above focused on one or a few CABs for specific study populations and used a limited scope of the study population. For example, the study by Mlambo et al. [1] studied one CAB on HIV community and it interviewed only the CAB members.

The strength of this study is that it employed mixed methods of data collection and analysis and it included the entire CAB membership. However, the study did not assess procedures followed when forming the CABs which is a limitation. Also, it would be useful to study views of trial participants about the role of CABs in their communities something that was not catered for by this study. Also, the qualitative component missed the views of regular CAB members which we think could be useful. The study also focused on training in ethics as a measure for capacity but there could be other ways of measuring the capacity of CABs. The other limitation is that we employed a cross sectional study and conducted interviews to assess the skills and abilities of CAB members but we were not able to observe or implement an intervention to assess how CABs perform their role. An intervention study to compare how CABs perform their role after training would be recommended because one can have the training but fail to perform due to several other challenges.

## Conclusions

The study shows that CABs in Uganda have some level of capacity to perform their roles. They are able to understand and review study documents and can offer guidance on community norms and expectations as well as giving valuable feedback to the investigators. Their functionality is however hindered by several challenges which include: lack of knowledge and training in research ethics, lack of resources to facilitate their work, lack of independence, and lack of guidelines for their operations. This study highlights the strengths of CABs and areas limiting their capacity which should to be addressed in order to achieve effective engagement of communities in health research.

## Supplementary Information

**Additional file 1.** Dataset.

**Additional file 2.** Data collection tools.

## Data Availability

The dataset supporting the conclusions of this article is included within the article (see Additional file 1). The data collection tools including the structured questionnaire and the KII guides are also included in the article (see Additional file 2). The transcripts for the qualitative component are available from the corresponding author on request.

## Abbreviations

CAB: Community Advisory Board
KII: Key informant interview
FGD: Focus group discussion
EDCTP: European and developing countries clinical trials partnership
HIV: Human immunodeficiency virus
IQR: Interquartile range
