# Supplementary material for: Capacity of community advisory boards for effective engagement in clinical research: a mixed methods study
Source: BMC Med Ethics. 2021 Dec 15;22:165. doi: 10.1186/s12910-021-00733-0 (PMC8672338; doi:10.1186/s12910-021-00733-0)
Supplement: Supplementary file 2 — Additional file 2. Data collection tools. [file 12910_2021_733_MOESM2_ESM.pdf]

Structured questionnaire used to collect quantitative data from CAB members and the KII guides used to collect qualitative data from trial investigators, CAB chairpersons and Community Liaison Officers.

Structured questionnaire: CAB Members

Brief introduction of the study

We are collecting data on past and existing community engagement structures in clinical trials. In particular, we aim to assess the existence and functionality of Community Advisory Boards (CABS) and conduct a needs assessment to identify capacity and training gaps of the CABs. The information you provide will inform the development of guidelines and training manual for CABs in the country.

No.	Questions and filters	Coding categories	Skips
	**Social demographics**		
1.	RECORD SEX OF RESPONDENT	1 Male 2 Female	
2.	How old were you on your last birth day?	|___|___| years	
3.	Have you ever attended school?	1 Yes 2 No	
4.	What is the highest level of school you completed?	1. Primary<br>2. Secondary<br>3. Tertiary/ University	
5.	What is your main occupation?	1. Formal employment<br>2. Farming<br>3. Business<br>4. Other (specify) --------------	
6.	What is your marital status?	1. Married / co-habiting<br>2. Widow/Divorced/Separated<br>3. Never married	
7.	How did you join the CAB?	1. Appointed by investigator<br>2. Nominated by the community<br>3. Nominated by the research institution<br>4. Recommended by a CAB member<br>5. Other (Specify) ---------------------	
8.	Which criteria is followed for a person to serve on the CAB?	1. Commitment to serve<br>2. Education level<br>3. Technical knowledge<br>4. Represent a specific population<br>5. Well known in the community<br>6. Known to the PI<br>7. Local leader/opinion leader<br>8. Other (specify) …………………	
9.	How long have you served on the CAB?	|___|___| Years	
10.	What is your role on the CAB?	1. Chairperson<br>2. Secretary<br>3. Member	
11.	Which category of population or community do you represent on the CAB?	1. Adolescents<br>2. General population<br>3. Mothers<br>4. Key population	

		5. Patient group(specify) ___________________<br>6. Vulnerable population<br>7. Other (specify) ____________	
12.	Have you been involved in protocol development process for any clinical trial?	1. Yes<br>2. No	
13.	Have you attended training on the protocol?	1. Yes<br>2. No	
14.	How many times have you attended these trainings in a year?	1. Once<br>2. Twice<br>3. Three or more times	
15.	Who initiated and facilitated the training?	1. Principal investigator<br>2. Cross-CAB network<br>3. Research Institution<br>4. On-line training<br>5. Regulatory body e.g. UNCST, REC<br>6. Others (Specify) ____________________	
16.	Do you sometimes encounter conflict of interest while serving on the board?	1. Yes<br>2. No	
17.	Where conflict of interest is involved, how is it resolved?	1. Meet with the PI over it<br>2. Report to REC<br>3. Declare it	
18.	For each of the statements I'm going to read out, please tell me if it is **TRUE, NOT TRUE OR DON'T KNOW.** CAB members are responsible for:<br><br>a) Informing research priorities based on community needs<br>b) Advising researchers on community norms and expectations<br>c) Contributing to an effective informed consent process by providing input and reviewing forms and questionnaires<br>d) Engaging in protocol development and review<br>e) Helping to build trust with the community<br>f) Creating a supportive environment for trials by raising awareness and dispelling myths about trial research<br>g) Facilitating recruitment by identifying contacts to relevant target groups	**True** **Not true** **Don't know**<br>1 2 2<br>1 2 3<br>1 2 3<br>1 2 3<br>1 2 3<br>1 2 3<br>1 2 3<br>1 2 3	

	h) Following up closely on the research processes<br>i) Conveying information about the research to the community.<br>j) Give feedback from the community to the researcher Conducting community sensitization<br>k) Analyzing findings from particular stakeholder perspectives<br>l) Assisting in the dissemination of results to appropriate audiences<br>**m)** Advising trial site on ways in which research practice can contribute to the improvement of local standards of life.	1 2 3<br>1 2 3<br>1 2 3<br>1 2 3<br>1 2 3	
19.	Have you attended any research ethics training?	1. Yes<br>2. No	
20.	If YES, which of the following modules were covered during the training?<br><br>READ OUT OPTIONS	Y N DK<br>Research ethics 1 2 3<br>Health research 1 2 3<br>Human subjects-protection 1 2 3<br>Responsible conduct-of research 1 2 3<br>Good Clinical Practice 1 2 3<br>Others- 1 2 3<br>(Specify)___________________________	
21.	Have you attended a training in community mobilization and engagement?	1. Yes<br>2. No	
22.	Does your CAB have guidelines that inform your operations?	1. Yes<br>2. No	
23.	Are the guidelines institutional or non-institutional (community, disease-specific or study based)?	1. Institutional<br>2. Non-institutional	
24.	Do you have a dedicated office/secretariat for CAB operations	1. Yes<br>2. No	
25.	Do you get facilitation to attend CAB meetings?	1. Yes<br>2. No	
26.	What kind of facilitation do you get?	1. Transport refund<br>2. Air time<br>3. Lodging<br>4. Allowance	
27.	How many CABs do you subscribe to?	|___| Number	
28.	Do you interact with trial participants?	1. Yes<br>2. No	

29.	During your tenure of office, how often have you interacted with the trial participants?	|___| Visits	
30.	During your interactions did you identify the following issues and how many times? READ OUT<br>a) Informed consent →<br>b) Transport refund →<br>c) Side effects →<br>d) Wrong study procedure →<br>e) Coercion to join the study →<br>f) Coercion to stay in the study →<br>g) Medical insurance →<br>h) Patient related injuries and admissions →	Frequency (times)<br>0 1 2 3 4+<br>0 1 2 3 4+<br>0 1 2 3 4+<br>0 1 2 3 4+<br>0 1 2 3 4+<br>0 1 2 3 4+<br>0 1 2 3 4+<br>0 1 2 3 4+	
31.	If yes to any **[for options *a-h*],** did you make any recommendations to the investigator?	1. Yes<br>2. No	
32.	If yes, did the investigator act on the recommendations?	1. Yes<br>2. No	
33.	If yes, what action(s) did the investigator take?	1. Made ammendments to the protocol<br>2. Reported to the REC<br>3. Stoppped enrollment<br>4. Reconsented participants<br>5. Filled out deviations/ violation report<br>6. Further training of the study team<br>7. Corrective actions reported back to the community by the investigator/CAB<br>8. Closed the study<br>9. Other (specify)-------------------------	

Key Informant guide: Trial Investigators

Title of the study:

Some background questions

i. Age
ii. Sex
iii. Place of work/ institution
iv. Occupation
v. Field of specialization
vi. Highest level of education attained
vii. Duration of service as investigator

Brief introduction of the study

We are collecting data on past and on existing community engagement structures in clinical trials. In particular, we aim to assess the existence and functionality of Community Advisory Boards (CABS) and conduct a needs assessment to identify capacity and training gaps of the CABS. The information you provide will inform the development of guidelines and training manual for CABs in the country.

Guiding Questions

1. What do you understand by community engagement in clinical trials?
2. What are the different types of community engagement approaches you know?
3. Share with us the different community engagement approaches you have used for the different trials you have been involved in and your experience working with them. (probe for aspects of establishment, facilitation, functionality and feedback mechanisms
4. Which community engagement approaches have worked and why? Which approaches didn't work and why?
5. Community advisory boards are one of the community engagement approaches. What is your opinion about their functionality and effectiveness?
6. What's your opinion about CAB's knowledge and understanding of:

   Prompts

   - local cultures and perspectives.
   - languages,
   - dynamics of local trials,
   - concerns of vulnerable or marginalized populations, and
   - local priorities.
7. How are the CABs established or formed? Are there any guidelines or procedures***?*** Are these guidelines followed as reference documents during the formation of the CABs (examples of

Guidelines: GPP for Biomedical Trials, 2011, *Prompts: appointments, membership requirements, SOPs, guidelines, manuals*

8. How are CAB members identified?
9. How are CAB members selected? How are their roles and tenure of office decided?

   Probe: *Representation, number, cultural insight, technical expertise, access and leadership potential, commitment to advance research, voluntarism, maintaining confidentiality and commitment to the CAB's mission, roles and responsibilities.*
10. How do Investigators ensure that CAB members perform their functions? Probe into: *trainings and refreshers courses for members in ethics and research conduct, commitment to the CAB, involving the members in decision making, being available to support the members,*
11. Have you conducted any training for your CAB? If yes please elaborate on the types or fields of the training, frequency, duration and mode of delivery.
    How are the CAB activities monitored and evaluated?
12. How often do you receive feedback from your CAB? What type of recommendations have you received (closure, amendment, termination etc…) and how do you act on their recommendations? How do you act on issues of violation of participant's rights and non-compliance?
13. Who regulates CABs activities? Prompts: *regulator, investigator, research institution, Monitoring, reporting, meetings, etc.*
14. What challenges do CABs face in performing their roles? What can be done to solve the challenges?
15. Community advisory board is an idea that was initiated in the western countries. We would like to get your opinion on whether this applies to the Ugandan local setting and if it serves its intended purpose. Do you think that the name CAB suits its role in the local context?
16. Currently CABs are established by PIs. In your opinion, what mechanisms do you recommend for regulating CABs?
17. Any other information you would like to share with us?

Thank you.

Key Informant guide: CAB Chairpersons

Brief introduction of the study

We are collecting data on past and on existing community engagement structures in clinical trials. In particular, we aim to assess the existence and functionality of Community Advisory Boards (CABS) and conduct a needs assessment to identify capacity and training gaps of the CABS. The information you provide will inform the development of guidelines and training manual for CABs in the country.

Some background questions

i. Age
ii. Sex
iii. Place of work/ institution
iv. Occupation
v. Field of specialization
vi. Highest level of education attained
vii. Duration of service as a CAB member

Guiding questions

1 In your opinion, what do you understand by community engagement in clinical trials?

2 Describe the methods you have used to engage with the communities to participate in research.

3 From your experience as a CAB member, what reasons do participants give for their participation and non-participation in clinical trials?

4 What is your role as a CAB member?

1. How did you become a CAB member? Are there any guidelines/ procedures/SOPs that define membership? Please explain.
2. As a CAB member, explain how your activities are monitored and regulated? By who, when, for what purpose, etc.
3. Have you attended any trainings/ refresher courses in research ethics and human rights protection in clinical trials? Probe into: types of trainings, duration, certification, frequency, who conducted the training, etc.
4. Have you ever raised any recommendations from the community to the investigator? How was it addressed?
5. Community advisory board is an idea that was initiated in the western countries. We would like to get your opinion on whether this applies to the Ugandan local setting and if it serves its intended purpose. Do you think that the name CAB suits its role in the local context? Should it change? Please propose

6. Currently CABs are established by PIs, in your opinion what mechanisms do you recommend for regulating CABs?
7. What is your opinion on the independence of the CABs to take decisions during a clinical trial
8. How have you performed your activities as a CAB member? Probe; *community entry, community education, consultations with the communities, consultation with the investigators, advising community on pertinent issues, etc.*
9. In your opinion as a CAB member, do you feel that your views in regards to community are considered by the PI?
10. How do you assess your role as a CAB member? (*no of meetings, contributions to society, recognition by UNCST, PI or the society, merit , invitation for trainings*)
11. Is there any other information you would like to share with us?

Thank you.

Key informant guide: Community leaders/Community Liaison Officers:

Brief introduction of the study

We are collecting data on past and on existing community engagement structures in clinical trials. In particular, we aim to assess the existence and functionality of Community Advisory Boards (CABS) and conduct a needs assessment to identify capacity and training gaps of the CABS. The information you provide will inform the development of guidelines and training manual for CABs in the country.

Some background questions

i. Age
ii. Sex
iii. Place of work/ institution
iv. Occupation
v. Field of specialization
vi. Highest level of education attained
vii. Duration of service as a community leader

Guiding questions

1. What is your role as a community leader prior, during and after conduct of a clinical trial?
2. What considerations do you think should be made when conducting clinical trials in the communities?
3. What do you think is the role of community members in clinical trials?
4. In your opinion, are community members adequately consulted and involved in clinical trials with in their communities? Probe: *How, when, by who, on what, for what period, etc*?
5. In your opinion do you think clinical research Investigators protect the rights of participants in clinical trials? If Yes how? If No why?
6. What should be done to ensure that communities are not exploited during clinical trials?
7. Is your community sensitized and educated on the different aspects of clinical trials being conducted? How, when, where
8. What challenges do you face as a leader in engaging the community during clinical trials? How can these challenges be solved?
9. Any other information you would like to share with us?

Thank you.
